# Structure of the Ty3/Gypsy retrotransposon capsid and the evolution of retroviruses

**DOI:** 10.1073/pnas.1900931116

**Published:** 2019-04-29

**Authors:** Svetlana O. Dodonova, Simone Prinz, Virginia Bilanchone, Suzanne Sandmeyer, John A. G. Briggs

**Affiliations:** ^a^Structural and Computational Biology Unit, European Molecular Biology Laboratory, 69117 Heidelberg, Germany;; ^b^Department of Molecular Biology, Max Planck Institute for Biophysical Chemistry, 37077 Gottingen, Germany;; ^c^Department of Biological Chemistry, University of California, Irvine, CA 92697;; ^d^Structural Studies Division, MRC Laboratory of Molecular Biology, Cambridge Biomedical Campus, CB2 0QH Cambridge, United Kingdom

**Keywords:** LTR retrotransposon, retrovirus, capsid, Gag, maturation

## Abstract

Retroviruses evolved from long terminal repeat (LTR) retrotransposons by acquisition of envelope functions, and subsequently reinvaded host genomes. Together, endogenous retroviruses and LTR retrotransposons represent major components of animal, plant, and fungal genomes. Sequences from these elements have been exapted to perform essential host functions, including placental development, synaptic communication, and transcriptional regulation. They encode a Gag polypeptide, the capsid domains of which can oligomerize to form a virus-like particle. The structures of retroviral capsids have been extensively described. They assemble an immature viral particle through oligomerization of full-length Gag. Proteolytic cleavage of Gag results in a mature, infectious particle. In contrast, the absence of structural data on LTR retrotransposon capsids hinders our understanding of their function and evolutionary relationships. Here, we report the capsid morphology and structure of the archetypal Gypsy retrotransposon Ty3. We performed electron tomography (ET) of immature and mature Ty3 particles within cells. We found that, in contrast to retroviruses, these do not change size or shape upon maturation. Cryo-ET and cryo-electron microscopy of purified, immature Ty3 particles revealed an irregular fullerene geometry previously described for mature retrovirus core particles and a tertiary and quaternary arrangement of the capsid (CA) C-terminal domain within the assembled capsid that is conserved with mature HIV-1. These findings provide a structural basis for studying retrotransposon capsids, including those domesticated in higher organisms. They suggest that assembly via a structurally distinct immature capsid is a later retroviral adaptation, while the structure of mature assembled capsids is conserved between LTR retrotransposons and retroviruses.

## Significance

Long-terminal repeat (LTR) retrotransposon sequences are widespread in eukaryotic genomes. They have been adapted to perform functions ranging from placental development to antiviral defense. Recently, a synaptic protein involved in memory, Arc, was shown to derive from a Ty3/Gypsy retrotransposon capsid. Retroviruses like HIV-1 are thought to have evolved from LTR retrotransposons by acquiring an envelope protein. Despite broad importance, we have lacked structural data on LTR retrotransposon capsids. Here, we determined the Ty3 capsid structure. We found striking similarity to mature HIV-1 capsids. HIV-1 assembles an immature virus particle that rearranges into a mature form. In contrast, Ty3 seems to directly assemble the mature form, suggesting retroviruses evolved their immature state to facilitate an extracellular step in the life cycle.

Retroviruses and long terminal repeat (LTR) retrotransposons share gene architecture, most commonly containing two overlapping ORFs: *GAG*, which encodes the structural proteins, including capsid (CA) and nucleocapsid (NC), and *POL*, which encodes enzymes, including protease (PR), reverse transcriptase (RT), and integrase. The most extensively studied LTR retrotransposon families, Ty3/Gypsy (1), Ty1/Copia (2), and Bel/Pao (3), are also classified as virus families: Metaviridae, Pseudoviridae, and Belpaoviridae, respectively. Based on similarities in their replication mechanisms and protein components, these families have recently been grouped together with the Retroviridae in the new order of reverse-transcribing viruses, the Ortervirales (4). Unlike the retroviruses, retrotransposons typically lack genes for envelope proteins and do not have an extracellular stage in their life cycle. It has been proposed that retroviruses diverged, possibly in more than one lineage (5), from the Ty3/Gypsy family of retrotransposons by acquisition of the *ENV* gene (6, 7), but their evolutionary relationships remain unclear. An ancestral ortervirus, encoding CA, PR, and RT, likely existed before plants and animals diverged (8).

Orterviruses have been exapted/domesticated to perform functions ranging from placental development to antiviral defense (9, 10). Interestingly, a neuronal gene, *Arc*, which appears to derive from a domesticated Ty3/Gypsy retrotransposon, encodes a bilobar capsid domain with structural similarity to retroviral CA proteins (11, 12). Arc was recently shown to form capsid-like structures, which are implicated in neuronal function and memory (13, 14).

The *GAG* gene in retroviruses and retrotransposons is initially expressed as Gag and Gag-Pol precursor polyproteins. Gag is greatly abundant over Gag-Pol and forms the structural basis of immature particle assembly and genome packaging (15, 16). In retroviruses, the conserved domains of Gag are MA (the matrix domain), which interacts with the viral membrane; CA, which oligomerizes to form the viral capsid; and NC, which packages the genome, while the presence of other domains or spacer peptides between or downstream of these domains varies among retroviruses. CA consists of two subdomains CA-NTD (N-terminal domain) and CA-CTD (C-terminal domain). High-resolution structures are available for multiple CA-NTD and CA-CTD domains, including HIV-1 (lentivirus), Rous sarcoma virus (an alpharetrovirus), Mason–Pfizer monkey virus (a betaretrovirus), and murine leukemia virus (MLV; a gammaretrovirus) (17–22). Gag assembles within the cytoplasm or at the plasma membrane into partial spheres formed by a curved, hexameric Gag lattice containing irregularly shaped gaps (23). These are subsequently released as immature membrane-bound particles. Activation of the viral PR leads to cleavage of Gag into its component domains. Upon proteolytic maturation, many CA–CA interactions are broken and new interactions are established. CA then reassembles around the condensed genome as a characteristic conical or polygonal fullerene capsid, which can be a closed shell or an incomplete or wrapped structure (24–27). Viral entry into the new host cell deposits the capsid and triggers the subsequent infection program. How the CA domains are arranged within immature and mature retroviral particles has been determined using cryo-electron tomography (cryo-ET) of in vitro assembled particles and of native virus particles for HIV-1, MLV, and other viruses (20, 26, 28–33). Despite sequence diversity, within the immature Gag lattice, the packing of the CA-CTD is largely conserved among retroviruses, while the CA-NTD arrangement is highly divergent. After maturation, the CA–CA interactions in the capsid are largely conserved. Maturation occurs within the limited space of the viral envelope, but the hexamer-hexamer spacing is larger in the mature virus (∼10 nm) than in the immature virus (∼8 nm). To accommodate this increased spacing, either only a subset of CA is incorporated into the mature core (e.g., HIV-1) (34, 35) or the mature core is a multilayered structure (e.g., MLV) (26).

Similar to retroviruses, most members of the Ty3/Gypsy family have Gag proteins (Gag3) that contain CA and NC domains but lack MA (1). Although members of the Ty1/Copia class can also encode NC, Ty1 itself does not. Ty1 and Ty3 Gag proteins, together with lesser amounts of Gag-Pol, form roughly spherical virus-like particles of variable sizes within cells (36–39) that also undergo proteolytic maturation. In the case of Ty3, Gag3 is cleaved into CA and NC domains (40). Formation of the capsid is important for genome protection and is an essential step in the retroelement life cycle.

The CA proteins of retroviruses and LTR retrotransposons have distant but detectable homology (5, 8). A low-resolution structure is available for assembled Ty1 capsids, but the structure may contain artifacts due to the imposed symmetry and cannot be interpreted in terms of CA domains (37, 38). Ty3 particles were studied by atomic force microscopy, which suggested an icosahedral capsid, but did not provide further structural details (36, 41). Beyond these studies, there is virtually no direct structural information about retrotransposon capsid arrangement.

Here, we have determined the structure and molecular architecture of the Ty3 capsid by 3D and 2D cryo-electron microscopy (cryo-EM) and compared it with those of the retroviruses. These comparisons have profound implications for our understanding of the evolution of retroviral lifecycles.

## Results

### Wild-Type and PR Mutant Ty3 Particles Observed Within the Cell by ET.

**Fig. 1.**
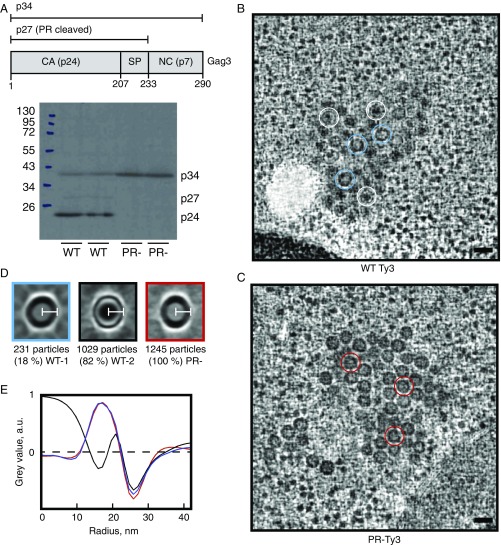
WT and PR- Ty3 particle morphology. (*A*) Schematic Ty3 Gag3 polyprotein showing regions corresponding to p34 (aa 1–290), p27 (aa 1–233), and CA p24 (aa 1–207). Western blot analysis of yeast cells expressing WT or PR- Ty3. Note that Gag3 (p34) and its PR cleavage products (p27, p24) are known to migrate anomalously (41). (*B*) Slices through representative tomographic reconstructions of resin-embedded yeast cells containing WT Ty3 particles. Representative WT type 1 particles (thick-ring morphology) are marked with blue circles, and representative WT type 2 particles (thin ring morphology) are marked with white circles. (Scale bar, 50 nm.) (*C*) Slices through representative tomographic reconstructions of plastic-embedded yeast cells containing PR- Ty3 particles. Representative PR- particles are marked with red circles. Particles are homogeneous, and all have a thick-ring morphology. (Scale bar, 50 nm.) (*D*) Central slices through the particle averages for WT-1, WT-2, and PR- Ty3 populations. (White scale bars, 21 nm.) (*E*) Radial profiles through the particle averages. The WT-1 and PR- particles with immature-like morphology have the same radius and radial profile, while WT-2 particles with mature-like morphology have the same radius but a different radial profile.

Expression of wild-type (WT) or PR mutant (PR-) Ty3 was induced in yeast cells from which endogenous copies of Ty3 had been deleted. The Ty3 expression and Gag3 cleavage state were confirmed by Western blot analysis (Fig. 1*A*). Cells were high-pressure-frozen, processed, embedded in Lowicryl resin, sectioned, and imaged in an electron microscope. Tomographic datasets were acquired for both WT and PR- Ty3 samples.

Upon visual inspection of the tomographic data, both WT and PR- Ty3 particles were readily identified within the cells and formed large, closely packed clusters (Fig. 1 *B* and *C* and Movies S1 and S2). While isolated retrotransposon particles can often be found in laboratory yeast strains, these large clusters are characteristic of cells overexpressing Ty3 and are not observed in Ty3-null cells (40). Among the WT Ty3 particles, two morphologically distinct types could be distinguished (Fig. 1*B* and Movie S1). The particles of the first type (18% of all WT particles; blue arrows in Fig. 1*B*) appear in section as thick dark rings and are empty on the inside, while particles of the second type (82% of all WT particles; white arrows in Fig. 1*B*) appear in section as thin rings with dark condensed material in the middle. These two particle types are reminiscent of the appearance of immature and mature retroviral particles in EM: In immature retroviruses, the immature, uncleaved Gag/ribonucleoprotein particle (RNP) layer appears as a thick shell, whereas the mature, cleaved CA appears as a thin layer containing a condensed RNP (42). The particles we observed in cells expressing PR- Ty3, in which Gag3 is uncleaved, exhibited exclusively the morphology of the first type (Fig. 1*C* and Movie S2), confirming that this represents the immature form.

We analyzed the WT and PR- Ty3 morphology in more detail by identifying all particles within the tomograms, cropping them out, and averaging them in three groups: WT type 1 (thick ring), WT type 2 (thin ring), and PR- (thick ring) (Fig. 1*D*). From the particle averages, we determined the radial density profile of the particles (Fig. 1*E*). WT type 1 and PR- particles have indistinguishable thick-ring radial density profiles, supporting the assertion that both represent Gag3 particles that have not undergone cleavage between CA and NC and are immature. Both immature and mature particles have the same external radius of ∼21 nm, corresponding to a true radius of ∼25 nm before Lowicryl embedding and beam exposure.

### The Architecture of PR- Ty3 Particles Determined by Cryo-ET and Subtomogram Averaging.

**Fig. 2.**
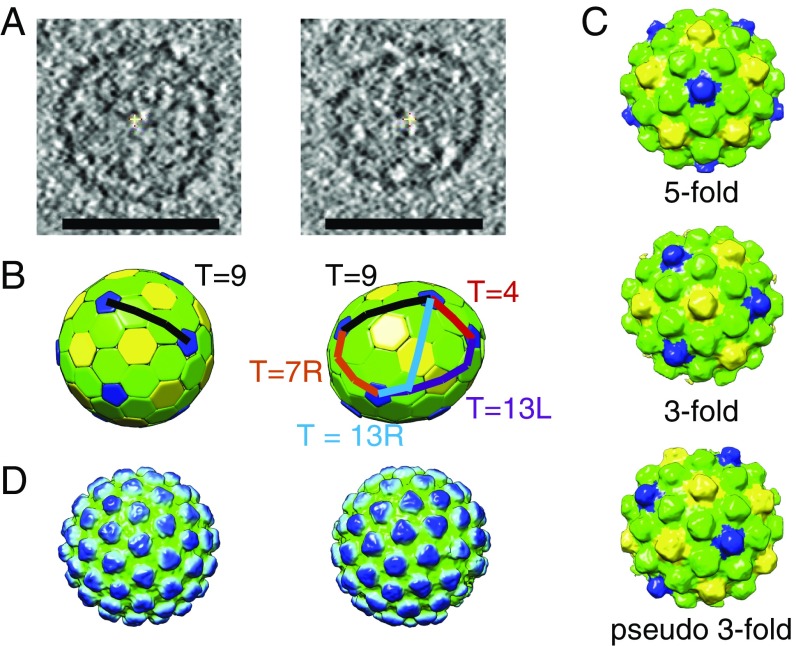
Cryo-ET of PR- Ty3 particles. (*A*) Slices through the tomographic reconstructions of purified, plunge-frozen PR- Ty3 particles (shown as an average of 10 computation slices). A regular icosahedral T = 9 particle (*Left*) and an irregular particle with a variable T-number (*Right*) are shown. (Scale bars, 50 nm.) (*B*) Ty3 lattice maps visualized by placing hexagons or pentagons at the positions of capsomers. Note the uniform distribution of pentamers in the T = 9 particle (*Left*) and the uneven distribution of pentamers in the other particle (*Right*). Vectors connecting neighboring fivefold positions are shown as lines, and local T-numbers are indicated. Fivefold positions are colored blue, threefold positions are colored yellow, and pseudothreefold positions are colored green. (*C*) Subtomogram averages of the fivefold, threefold, and pseudothreefold positions within the Ty3 PR- particles. Within each structure, the fivefold position is colored blue, the threefold positions is colored yellow, and the pseudothreefold position is colored green. (*D*) Composite representation of complete Ty3 particles, colored radially from green (low radius) to blue (high radius).

Next, we wanted to study the structure of the Ty3 particle capsid in more detail. We lysed the PR- expressing cells and purified the Ty3 particles by sucrose density gradient according to the protocol described by Kuznetsov et al. (41) (*SI Appendix*, Fig. S1). We were unable to reliably purify WT particles due to their reduced stability and the presence of a mixture of immature and mature particles. The purified Ty3 PR- particles were plunge-frozen and subjected to cryo-ET (Fig. 2*A*).

We identified 148 particles, extracted subtomograms along their surfaces, and subjected them to reference-free subtomogram averaging analysis. After several alignment iterations, an approximately sixfold symmetrical preliminary structure of the particle surface was obtained (*SI Appendix*, Fig. S2). We placed a hexameric object at the positions and orientation of all subtomograms found during the alignment procedure; in this way, we displayed a “lattice map” showing the positions of capsomers in the capsid (Fig. 2*B*). Capsomers could be distinguished according to whether they were fivefold coordinated (blue), sixfold coordinated next to a fivefold position (pseudothreefold, green), or sixfold coordinated surrounded by sixfold positions (true threefold, yellow).

These three groups were separately aligned and averaged (Fig. 2*C*) and then combined at appropriate positions to generate low-resolution reconstructions of individual PR- Ty3 particles (Fig. 2*D*). Capsomers at fivefold positions appeared pentameric, while those at sixfold coordinated positions appeared trimeric (Fig. 2*C*).

Visual inspection of multiple lattice maps showed that while a large fraction of particles [106 of 148 (72%)] are damaged or incomplete, 28% (42 of 148) of the imaged particles are complete closed structures. Complete closed structures always contained 12 pentamers, consistent with the requirements of fullerene geometry. Next, the triangulation numbers (T-numbers) of the Ty3 capsids were calculated. T-numbers define the relative positions of pentamers and hexamers on the capsid surface (43) (details are provided in *Materials and Methods*). In total 13% (19 of 148) of all particles had mixed T-numbers (including 3, 4, 7, 12, 13, and 16), meaning that the pentamers are not uniformly distributed over the particle surface (Fig. 2*B*, *Right*). Mixed T-numbers lead to deviation of the particle shape from spherical toward more elliptical and irregular [Fig. 2 *A*, *B*, and *D* (*Right*) and *SI Appendix*, Fig. S3]. We found that 39 of the 42 complete Ty3 particles were T = 9 icosahedra, where two nearest pentamers in the lattice are always separated by two hexamers sitting along one vector (Fig. 2*B*, *Left* and *SI Appendix*, Fig. S3). The other complete particles had mixed T-numbers.

Immature PR- Ty3 Gag can therefore assemble “closed” particles. Both incomplete and closed particles contain pentamers that may be unevenly distributed (mixed T-number). This arrangement is unusual and contrasts with typical icosahedral virus capsids, which have uniformly distributed pentamers. It also contrasts with immature retroviruses, which lack pentamers and always form incomplete spheres containing irregularly shaped gaps (23). Instead, it is more similar to the mature capsids of retroviruses such as HIV-1, which include pentamers and form both incomplete and closed structures with unevenly distributed pentamers, giving locally variable T-numbers (16, 24).

### The Structure of PR- Ty3 Particles Determined by Single-Particle Cryo-EM.

**Fig. 3.**
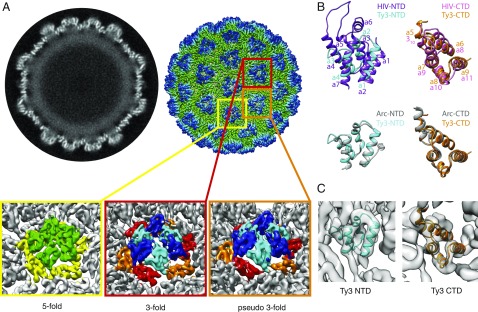
Cryo-EM reconstruction of a T = 9 PR- Ty3 particle. (*A*, *Left*) Slice through the center of the PR- Ty3 particle reconstruction resolved at 7.5 Å. The outer layer with distinct α-helical densities is the CA layer, and the fainter blurred layer underneath likely represents spacer, NC, and the associated genome. (*A*, *Right*) Three-dimensional reconstruction of a PR- Ty3 particle colored radially from green (low radius) to blue (high radius). (*Insets*) Close-up views of the fivefold (yellow box), threefold (red box), and pseudothreefold (orange box) positions. Within the positions with different symmetries, the densities are colored to indicate the different domains of CA: Fivefold CA-NTD is colored green, CA-CTD is colored yellow, threefold and pseudothreefold CA-NTD is colored cyan/blue in conformation A/B, and CA-CTD is colored orange/red in conformation A/B. (*B*) Ty3 CA-NTD (cyan) and Ty3 CA-CTD (orange) homology models are shown superimposed on the HIV CA-NTD (purple; PDB ID code 4XFX) and HIV CA-CTD (pink; PDB ID code 4XFX) structures (*Upper*, in which helices are numbered) and the templates used for homology modeling: Arc N-terminal lobe (gray; PDB ID code 4X3I) and Arc C-terminal lobe (gray; PDB ID code 4X3X), respectively (*Lower*). (*C*) Rigid body fitting of the Ty3 homology models of the CA-NTD (cyan) and CA-CTD (orange) into the Ty3 cryo-EM map.

To study the structure of PR- Ty3 particles at higher resolution, we collected a 2D cryo-EM dataset of the purified particles, identified the icosahedral particles by image classification, and determined their structure to 7.5-Å resolution using the RELION (44) single-particle processing pipeline (Fig. 3*A* and *SI Appendix*, Fig. S4*A*). The particles have a radius to the center of the CA layer of ∼24 nm. The location of pentamers confirmed the T = 9 symmetry identified by cryo-ET and subtomogram averaging. The reconstruction showed clear α-helical densities consistent with the determined resolution, as well as a disordered internal layer (Fig. 3*A*), which may correspond to NC and associated nucleic acid.

To interpret the capsid architecture, a pseudoatomic model of the Ty3 capsid was required. A sensitive homology search performed with the HHpred server (45) identified the Arc N-terminal lobe and C-terminal lobe [Protein Data Bank (PDB) ID codes 4X3I and 4X3X] (11) as the best available templates, and these were used to generate a homology-based model of the Ty3 CA-NTD and CA-CTD, respectively (Fig. 3*B* and *SI Appendix*, Fig. S5*A*). In these models, the Ty3 CA-NTD is a bundle of four α-helices, while CA-CTD consists of five α-helices, consistent with the secondary structure predictions (*SI Appendix*, Fig. S5*B*).

The Ty3 homology models were fitted as rigid bodies into the EM map and showed excellent correlation with the EM map (Fig. 3*C*). Similar to retroviruses, the protruding capsomers of the Ty3 capsid are formed by the CA-NTD, while the inner layer, linking the capsomers together, is formed by the CA-CTD.

Consistent with the principles of virus architecture described by Caspar and Klug (43), the T = 9 Ty3 particle capsid is formed from 540 copies of CA. There are nine different (non–symmetry-related) copies of CA (one in the fivefold, two in the threefold, and six in the pseudothreefold positions within the complete Ty3 particle). Comparing the nine different non–symmetry-related positions, the fold of the individual CA-NTDs and CA-CTDs does not change, but their relative orientations change. Notably, the trimeric appearance of the sixfold coordinated positions in the EM map is a result of adjacent CA-NTDs existing in two very different orientations [Fig. 3*A*, orientation A (cyan) and orientation B (dark blue)].

### A Structural Model for the PR- Ty3 Capsid.

**Fig. 4.**
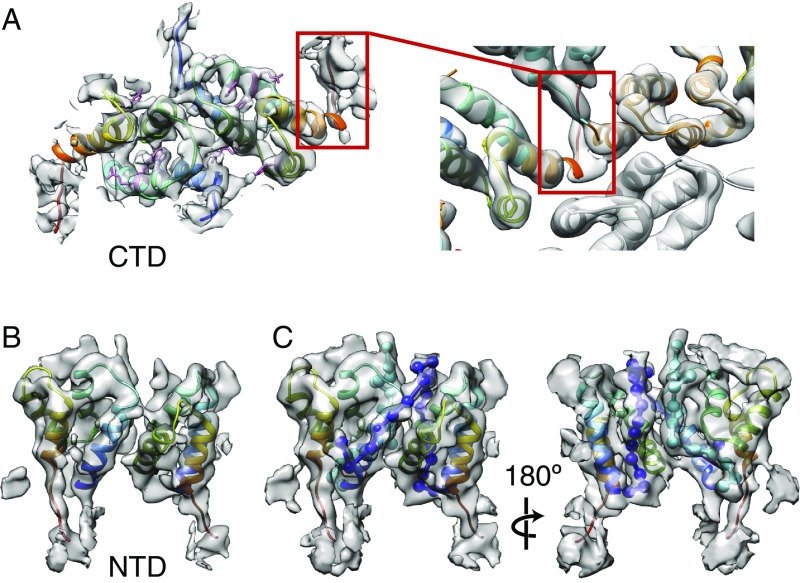
Ty3 CA-NTD and CA-CTD at higher resolution. (*A*, *Left*) Structure of a Ty3 CA-CTD dimer at 4.9 Å generated by alignment and averaging of nine non–symmetry-related copies of the Ty3 CA-CTD. The homology models of the Ty3 CA domains have been flexibly fitted into the refined EM maps. Protein models are colored from blue (N terminus) to red (C terminus). The bulky side chains of F134, R135, W138, R157, and Y164 amino acids in the CA-CTD are colored pink. (*A*, *Right*) Close-up view of the very C-terminal part of the CA-CTD from the complete Ty3 particle map, which creates one of the contacts between two neighboring CA monomers in the particle lattice. (*B*) Structure of a CA-NTD dimer at 5.5 Å generated by alignment and averaging of four non–symmetry-related copies of the Ty3 CA-NTD dimer, colored as in *A*. (*C*) As in *B*, but shown at a lower isosurface level to illustrate the unoccupied densities in the refined EM map of the CA-NTD dimer. These represent the very N-terminal parts of the Ty3 CA-NTD. The N-terminal part of the CA-NTD is shown as a string of beads in cyan (conformation A) and blue (conformation B). In both conformations, the N-terminal part of the protein first runs outward along the inner interface between the CA-NTD helix 1 and helix 2 (*C*, *Right*). In conformation B, it then continues over the top of the CA-NTD and down the outer side of the threefold position (*C*, *Left*).

We aimed to increase the resolution of the EM densities by averaging the non–symmetry-related capsomers (*SI Appendix*, Fig. S6 and Movies S3 and S4). Since the relative orientations of the individual domains differ within the capsid, we considered CA-CTDs and CA-NTDs separately. The densities for the nine non–symmetry-related copies of the Ty3 CA-CTD were aligned and averaged to generate a higher resolution 4.9-Å map (Fig. 4*A*, *SI Appendix*, Fig. S4*B*, and Movie S4). The homology model of the Ty3 CA-CTD (Fig. 3*B*) was then flexibly fitted into the map and showed very good agreement with the density (Fig. 4*A* and Movie S4). We observed protrusions from the EM densities around the α-helices, at positions corresponding to the large side chains (F134, R135, W138, R157, and Y164), confirming the quality of the model and the fit (Fig. 4*A*). We were also able to trace the very C-terminal short part of the CA-CTD, which appears to interact with the neighboring CA-CTD (Fig. 4*A*). The mutations (E190A/R191A) in that region cause a strong phenotype in yeast (46). These mutant Ty3 particles form long filaments in cells, instead of spherical particles, indicating that the C-terminal part of Ty3 CA-CTD is important for the capsid assembly. The positions of other residues where mutation has been previously described to disrupt particle formation, such as D60A/R63A or E148A/K149A (46), suggest that the phenotype may result from disruption of the domain structure rather than CA–CA interactions (*SI Appendix*, Fig. S7).

**Fig. 5.**
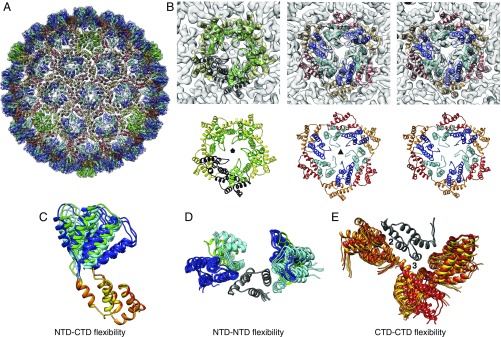
Variability of Ty3 CA-NTD and CA-CTD relative orientations. (*A*) Model of a complete Ty3 PR- particle showing fitted protein models and EM density (transparent gray). Proteins are colored as in Fig. 3. (*B*) Close-up views of the different positions within the Ty3 particle. One monomer in a fivefold position is highlighted in black for clarity. (*C*) Superimposition of the nine independent copies of CA showing the large relative movements of the CA-NTD and CA-CTD about the flexible interdomain linker. The CA-CTD was used for alignment of the structures. (*D*) Superimposition of the nine independent copies of CA-NTD, together with their neighbors, showing three distinct relative orientations aligned on one CA-CTD (gray). (*E*) Superimposition of the nine independent copies of CA-CTD, together with their neighbors, aligned on one CA-CTD (gray), illustrating continuous variability of the dimer and trimer CA CTD–CTD interfaces.

Adjacent CA-NTD domains exist in different orientations (Fig. 3*A*); therefore, we considered a pair of CA-NTDs [one in orientation A (cyan) and one in orientation B (dark blue)] to be the repeating unit, and aligned and averaged the four independent copies of this pair of CA-NTDs (excluding the CA-NTDs from the fivefold position) (*SI Appendix*, Fig. S6 and Movie S3). In this way, we generated a higher resolution 5.5-Å map of the CA-NTDs (Fig. 4*B* and *SI Appendix*, Fig. S4*B*). We flexibly fit the homology model of the Ty3 CA-NTD into the new EM map (Fig. 4*B*). The N-terminal 36 amino acids of the CA-NTD do not have a defined secondary structure in the homology model; however, in our map, we resolve density corresponding to this region. This density runs outwards along the interface between helix 1 and helix 2 of the CA-NTD in the middle of the threefold (and fivefold) position for ∼60 Å (Fig. 4*C* and Movie S3, cyan and blue densities). Interactions between these N-terminal parts of CA-NTDs may contribute to stabilization of the structure at threefold and fivefold positions (Fig. 5*B*, central densities). In orientation B, this density continues over the top of the CA-NTD and down the interface of helix 3 of one CA-NTD and helix 1 of the neighboring CA-NTD on the outer side of the threefold position (Fig. 4*C*, blue density). The total length of the density in orientation B in the EM map is ∼100 Å and likely accommodates all 36 amino acids. In conformation A, the N-terminal half of the density is not visible; it is not bound to the rest of the CA-NTD and is probably disordered. Instead, the surface of CA-NTD helix 3, where it would otherwise be bound, is occluded by binding the neighboring CA-NTD. The structure of the CA-NTD within the fivefold position is more similar to that of orientation A.

To generate a structural model for the complete PR- Ty3 capsid, we placed the models for the Ty3 CA-NTD and CA-CTD from flexible fitting back into all nine independent positions within the complete EM map and modeled the linker connecting the CA-NTD and the CA-CTD independently for all nine copies using Modeler (47). The complete fit is shown in Fig. 5 *A* and *B* and Movie S5.

### Variability of CA Structure Within the Capsid.

To assess the conformational variability of CA within the capsid, we superimposed and compared the nine independent copies of CA, showing large relative movements of the domains about the flexible interdomain linker (Fig. 5*C*). We also assessed the variability of CA CTD–CTD and CA NTD–NTD interactions by superposition of the different conformations. As expected, the main differences in CA-NTD orientation and interactions correspond to the differences between the A and B conformations, and interactions could be grouped into three distinct relative orientations corresponding to the observed A–B, B–A, and A–A (at the fivefold position) interactions (Fig. 5 *C* and *D*). The orientation and interactions formed by the CA-NTD within the fivefold position superimpose most closely with those of conformation A (Fig. 5*D*, green and cyan models). CA CTD–CTD interactions at the dimeric and trimeric interfaces showed a continuum of differing relative orientations (Fig. 5*E*).

The flexibility in conformation and orientation that we observe for CA allows formation of both fivefold and sixfold coordinated positions, as required to close an icosahedral capsid. Mature HIV-1 CA also shows conformational flexibility, allowing it to adapt to different local curvatures within the conical HIV-1 core and to form pentamers (30). Interdomain flexibility is therefore a conserved property of both retrotransposon and retroviral CA proteins.

### Comparison of the Ty3 and HIV-1 Capsids.

The Ty3 capsid is formed from 540 copies of CA, and has an interior volume of ∼5 × 10^4^ nm^3^. Assuming that two copies of the 5.2-kb Ty3 genome are packaged (48), this corresponds to ∼20 bases of genomic RNA per Gag molecule, and an RNA density of approximately one base of genomic RNA per 5 nm^3^ within the capsid. HIV-1 packages a dimeric 9.8-kb genome, and is formed from ∼2,400 copies of Gag (49), of which roughly half contribute to the mature capsid core (30). This corresponds to approximately eight bases of genomic RNA per Gag molecule in HIV-1. After maturation, the core has a volume of ∼2 × 10^5^ nm^3^ (50), giving an RNA density of approximately one base of genomic RNA per 10 nm^3^ within the HIV-1 capsid. Ty3 therefore packages significantly more RNA per copy of Gag, and per core volume, than HIV-1, but less than other orders of icosahedral ssRNA viruses (51).

**Fig. 6.**
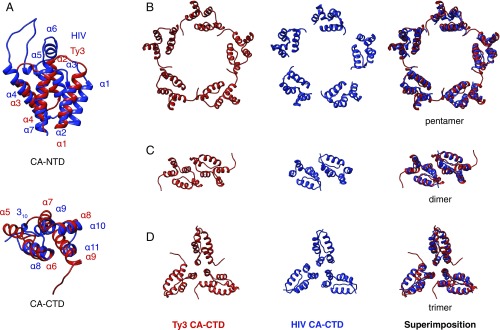
Structural comparison of the Ty3 and HIV capsid arrangements. (*A*) Superimposition of the Ty3 CA domains (red) and HIV-1 CA domains (blue). The α-helices are numbered. (*B*) Comparison of the fivefold CA-CTD quaternary structure of PR- Ty3 (*Left*) and mature HIV-1 (PDB ID code 3P05) (*Center*), and superposition of the two (*Right*). Note the high similarity of the arrangements. (*C*) Comparison of the dimeric CA-CTD arrangement of PR- Ty3 and mature HIV-1 (PDB ID code 4XFX). (*D*) Comparison of the trimeric CA-CTD arrangement of PR- Ty3 and mature HIV-1 (PDB ID code 4XFX).

Sequence comparison suggests that the structure of CA is conserved between retroviruses and Ty3/gypsy transposons, and that this conservation extends to caulimoviruses and pseudoviruses (8). We next directly compared the structures of the CA-NTD and CA-CTD domains in the PR- Ty3 capsid with those of HIV-1, confirming that they are highly conserved (Fig. 6*A*). This structural comparison corresponds very closely to the recent alignment by Krupovic and Koonin (8). Both CA-NTD and CA-CTD of Ty3 have the same core fold formed by four α-helices (Fig. 6*A*, *Right*); this “CA fold” is shared by the SCAN domain, which is a cellular fold likely exapted from a retrotransposon (52). CA (CA-NTD and CA-CTD together) therefore constitutes a double-SCAN domain fold. In HIV-1, this SCAN domain or CA fold is conserved, but two additional helices have been obtained within the CA-NTD (11). Helices 1–4 (the CA fold) in the Ty3 CA-NTD correspond to helices 2–4 and 7 in the HIV-1 CA-NTD (Fig. 6*A*, *Left*). Helix 1 in HIV-1 is found at the equivalent position to the N-terminal extension in Ty3. Helix 5 in the Ty3 CA-CTD corresponds to the 3_10_ helix in the HIV-1 CA-CTD, while helices 6–9 in Ty3 (the CA fold) correspond to helices 8–11 in HIV-1 (Fig. 6*A*, *Right*). We note that the structure of the foamy virus CA protein is also similar to that of Ty3, although foamy viruses have disordered stretches at the positions corresponding to Ty3 helices 3 and 8 (53).

The capsid of orthoretroviruses contains a conserved region in the CA-CTD called the major homology region (MHR) (*SI Appendix*, Fig. S5*C*), mutation of which causes defects in virus assembly and maturation (54). An MHR was previously identified within the Ty3 CA based on sequence comparison and mutagenesis (55, 56), but direct comparison of the HIV-1 CA structure with the model for the structure of Ty3 CA determined here shows that the proposed Ty3 MHR is not structurally equivalent to that in retroviruses, and previous homology models for the Ty3 capsid are incorrect (46).

We next compared the quaternary packing of CA within the assembled capsids of PR- Ty3 and HIV-1. In Ty3, the distance between the centers of neighboring hexamers is ∼11 nm, similar to that in mature HIV-1 capsids (∼10 nm) and larger than that in immature HIV-1 particles (∼8 nm). Strikingly, the quaternary arrangement of the PR- Ty3 CA-CTDs is almost identical to that of the CA-CTD in mature HIV-1 capsids at the level of dimer, trimer, and even pentamer (Fig. 6 *B*–*D*). Despite the similarity in the CA-CTD quaternary packing, there is very little conservation of residues at protein–protein interfaces. The CA-NTD packing is not well conserved (*SI Appendix*, Fig. S8), nor is the CA NTD–CTD interface that stabilizes the HIV-1 capsid (57) (*SI Appendix*, Fig. S9).

## Discussion

The CA proteins of metaviruses, like the Ty3 LTR retrotransposon, and of retroviruses, like HIV-1, have conserved “double-CA folds.” The conservation of this fold across the Ortervirales suggests that it was already present in an ancestral virus or transposon ∼1.6 billion y ago (8, 52). Here, we have observed that the degree of conservation extends beyond the fold. We have shown that the quaternary packing of the CA-CTD within the viral capsid is strikingly conserved between Ty3 and HIV-1. Furthermore, the architecture of the capsid is conserved: The Ty3 capsid and the HIV-1 capsid are both fullerene structures formed from pentameric and hexameric capsomers, where the pentamers can be unevenly distributed. The only substantial structural divergence is seen in the CA-NTD. Divergence in this region is not surprising, since it is the region directly exposed to host cytosolic factors, as well as the region that may also regulate access to the capsid interior through pores in the hexamers or pentamers.

Importantly, the immature, PR- Ty3 capsid does not share a conserved structure and architecture with the immature, PR- HIV-1 capsid but, instead, with the mature, processed HIV-1 capsid (28). How should this surprising result be interpreted? Retroviral and retrotransposon capsids provide a protected environment within the host cell cytoplasm within which to carry out nucleic acid metabolism. The initial assembly process is driven by the uncleaved Gag protein; this ensures that the viral genome is recruited and encapsidated simultaneously with assembly, and allows Gag–RNA–Gag interactions to contribute to assembly. Subsequent proteolytic cleavage between CA and NC releases the RNP, which becomes condensed into the center of the capsid, a step that may facilitate metabolism. This “RNP maturation” process is conserved between Ty3 and HIV-1. In addition to RNP maturation, retroviral capsids undergo dramatic “structural maturation.” The capsid disassembles to a large extent, and in the case of HIV-1, reassembles into its mature form using only a subset of the available CA protein. The CA hexamer-hexamer spacing is larger in mature retroviruses than in immature retroviruses, meaning that structural maturation without disassembly would lead to significant capsid expansion. In the case of Ty3, however, there is no change in particle size upon proteolytic cleavage, and therefore presumably no dramatic structural maturation. There are currently no structural data on assembled spumavirus capsids, but transmission EM suggests that the preassembled cytosolic capsids and packaged capsids have a similar size (31): Foamy viruses may also lack a structural maturation step. We therefore propose that Ty3 directly assembles a mature-like capsid, and that, upon proteolytic cleavage, the major structural changes are limited to RNP maturation.

Two evolutionary routes can be proposed: Ty3 has lost its immature capsid stage, evolving from an ancestral form that underwent a retrovirus-like capsid maturation, or, much more likely, retroviruses have evolved the immature stage from an ancestral virus that directly assembled a mature-like state. In Ty3, which lacks an extracellular stage in the life cycle, cleavage between CA and NC and resulting RNP maturation take place in the cytoplasm and require the protective environment of the capsid to prevent the RNP being exposed to host defense mechanisms during reverse transcription. Similarly, interactions between CA proteins need to be maintained during maturation to prevent CA diffusing away. In contrast, in HIV-1, RNP maturation occurs within an enveloped virus particle, where the limiting lipid membrane protects the genome and maintains the local capsid concentration. There is therefore no requirement to maintain an intact capsid during HIV-1 maturation. The ancestral protective function of the retroviral capsid is only needed after maturation and entry into a target cell. We therefore propose that the evolution of an enveloped extracellular stage in the retroviral life cycle freed the immature capsid from the selective pressures on the mature capsid, allowing the immature capsid structure to diverge to facilitate virus assembly and budding. Consistent with this hypothesis, the immature capsid structure is divergent among retroviruses.

The immediate early neuronal protein Arc, which is involved in multiple synaptic functions, including long-term potentiation (58), is a domesticated Ty3/gypsy retrotransposon (11, 13). Arc is structurally homologous to Ty3 CA. Intriguingly, the Arc protein has recently been shown to have retained its ability to form capsid-like structures (13), and capsid formation appears to be required for its neuronal function. There is intense interest in the structure and function of Arc capsids. Considering the high degree of conservation of quaternary CA interactions among the Orterviridae, the Arc capsid is likely to be structurally similar to the Ty3 capsid studied here.

The Ty3/Gypsy, Ty1/Copia, Bel/Pao, and Retroviridae family have been predicted to have homologous CA domain folds. Our data show that not only the fold but also the quaternary arrangement of CA-CTD in the capsid and the architectural principles of the capsid are conserved between Ty3 and retroviruses. We suggest that these are conserved ancient properties that will be found throughout LTR retrotransposons, retroviruses, and domesticated retrotransposons that form capsids.

## Materials and Methods

### Ty3 Expression and Yeast Cell Growth.

*Saccharomyces cerevisiae* strain yVB1680 (Ty3-null [ygrwty3-1 Δ::loxP, yilwty3-1 Δ::loxP], killer minus [L-A(−) L-BC(−)]) was derived from BY4741 (MATa *his3Δ1 leu2Δ0 met15Δ0 ura3Δ0*) (4040002; American Type Culture Collection). Killer double-stranded RNA particles are highly expressed in many laboratory strains (59), and could contaminate Ty3 particle preparations. The yVB1680 was therefore cured of killer L-A and L-BC double-stranded RNA particles (59) as described (60). The *YGRWTy3-*1 and *YILWTy3-1* were deleted by replacement by loxP-flanked selectable markers, followed by recombination mediated by transient CreA expression.

Yeast cells were grown in either complete medium (1% yeast extract, 2% peptone, 2% dextrose) or synthetic dextrose medium. Synthetic dextrose medium contained 0.67% yeast nitrogen base, 2% dextrose, complete amino acids, inositol, and adenine sulfate. For selection and growth of cells transformed with plasmids containing particular prototrophic markers, synthetic dextrose medium lacked selection nutrients.

WT or PR- Ty3 was expressed as described (41) from plasmids transformed into *S*. *cerevisiae* strain yVB1680 (strain BY4741 killer minus, Ty3 null). WT Ty3 expression was from plasmid pDLC201 (61). PR- Ty3 expression was from plasmid pJK776 that contains the catalytic core mutation D591 abolishing protease activity (62). Transformed cells were grown at 26 °C in synthetic raffinose medium [0.67% yeast nitrogen base, 1% raffinose, 2% (vol/vol) glycerol, 2% (vol/vol) sodium lactate] containing complete amino acids and adenine sulfate lacking selection nutrients to OD_600_ = 0.06. To induce Ty3 expression, galactose was added to a final concentration of 2%; cells were grown for 18 h and harvested at OD_600_ = 8–9.

### Immunoblot Analysis.

Whole-cell extracts (WCEs) were prepared by vortexing the cell suspension with glass beads in denaturing buffer (9 M urea, 5 mM EDTA). WCEs were fractionated and analyzed by SDS/PAGE. Proteins were transferred to nitrocellulose membrane and incubated with primary rabbit polyclonal anti-Ty3 CA antibodies (63) diluted 1:10,000. Proteins were visualized with horseradish peroxidase-conjugated secondary antibody.

### Yeast Cell Section Preparation and Cellular ET.

Ty3-expressing yeast cells were prepared for ET as described by Kukulski et al. (64). The yeast cell paste was high-pressure-frozen (Empact 2; Leica), processed by freeze substitution, and embedded in Lowicryl resin using an AFS2 (Leica). The samples were sectioned and mounted onto EM grids. Tomographic data were collected using an F30 Tecnai microscope (FEI) equipped with an Eagle CCD camera (FEI) with a pixel size at the specimen level of 11.8 Å. Dual-axis tilt series were collected with a 1° increment in a ±60° range. Tomograms were reconstructed in IMOD (65).

### Subtomogram Averaging of Particles from Cellular Tomograms.

Amira software (FEI) was used for tomogram visualization. Particles were manually picked, extracted, and translationally aligned and averaged using the TOM and Av3 software packages (66, 67). A total of 1,245 particles were picked and averaged from the PR- Ty3 cellular tomograms. All particles were homogeneous and had a “thick-ring” morphology. The WT tomograms contained a mixture of mature (*n* = 1,029) and immature (*n* = 231) Ty3 particles. The particles from the two classes were averaged separately. Each average was rotationally averaged to generate the radial density profile, and the particle radius was measured as 21 nm to the outside of the density layer in all cases. The diameter of the mature particles measured to the peak CA density was 40 nm, corresponding to a true diameter of ∼47 nm once sample shrinkage during embedding and imaging is considered. (Shrinkage has been estimated by measurement of equivalent structures in yeast samples prepared according to the protocol used here and in yeast samples prepared in vitreous ice.)

### Ty3 Particle Isolation.

Yeast cells grown to log phase were washed, and their cell walls were digested with zymolyase at 26 °C. The digestion was stopped by addition of 5 mL of ice-cold buffer A [10 mM trisaminomethane (Tris; pH 7.8), 1 mM EDTA, 5 mM NaCl] and 1 mM phenylmethane sulfonyl fluoride (PMSF). Lysed spheroplasts were pelleted for 5 min at 2000 × *g*. The pellets were washed twice with 3–5 mL of buffer A and PMSF, and were resuspended in 0.6 mL of cryobuffer [3 mM DTT, 1 mM PMSF, protease inhibitors (Sigma)]. The spheroplasts were vortexed with 0.5 g of glass beads at 4 °C. After a quick spin, the supernatant was layered on top of a 20%/30%/70% sucrose gradient and was fractionated for 2 h at 30,400 rpm in a SW55Ti rotor (Beckman) at 4 °C. Fractions enriched for Gag3 protein (*SI Appendix*, Fig. S1) were used for further sample purification. The particles were pelleted for 45 min in a SW60Ti rotor (Beckman) at 50,000 rpm, and the pellet was resuspended in the buffer containing 10 mM Tris (pH 8), 100 mM NaCl, and 1 mM EDTA. This washing step was repeated a total of three times. The resulting sample was used for cryogrid preparation. The protocol is also described in detail by Kuznetsov et al. (41).

### Cryogrid Preparation.

Purified Ty3 particles were applied to glow-discharged, copper, C-flat, carbon-coated grids. Grids were blotted for 12 s from the backside and then plunge-frozen into liquid ethane using a manual plunging device. Cryogrids were stored at liquid nitrogen temperatures until use.

### Three-Dimensional EM of Purified Ty3 Particles.

The grids containing purified Ty3 particles were imaged in a Titan Krios (FEI) electron microscope operated at 200 kV, equipped with a GIF 2000 CCD camera (Gatan). For ET, tilt series were collected with a 4.3-Å pixel size and a total dose of 60 electrons/Å^2^. The angular range was ±60° with a 3° increment. The range of defocus values was −3.5 to −6.5 μm. Fourteen tilt series were used for further processing. The tomograms were reconstructed in IMOD and later visualized in Amira. Subtomogram averaging was performed essentially as described previously (68) with the use of TOM and Av3 packages. The particles (148 particles in total) were picked manually from the tomograms and were split into two halves (even and odd based on the sequential number of the particle), which were processed completely independently. Initial positions for subtomogram extraction were evenly distributed on the surface of a sphere defined by the radius of each Ty3 particle. Subtomograms were extracted from the surface of the particles and were averaged together to produce the starting reference. Initial alignments were performed without symmetry application. After several iterations, the structure showed sixfold symmetry features (*SI Appendix*, Fig. S2). References were recentered at the sixfold axis, and several alignment iterations were performed with the applied C6 rotational symmetry. We visualized the particle lattice maps in Amira and Chimera (69), and noticed that the maps contained both sixfold and fivefold positions. Based on their geometric relation to the previously defined sixfold subunits, positions of pentamers were defined, followed by subtomogram extraction, averaging, and iterative alignments. Within the fivefold subtomogram average, an apparent threefold symmetry of the other capsomers became clear (Fig. 2*D*).

At that point, we classified subtomograms based on their position in the lattice. We generated subtomogram averages for three classes: fivefold (1,497 subtomograms), threefold (3,564 subtomograms), and pseudothreefold (6,724 subtomograms). The pseudothreefold positions are surrounded by five other threefold positions and by one fivefold position; thus, they are only pseudosymmetrical. Final reconstructions of the three classes (Fig. 2*D*) had a resolution of 22–23 Å. Upon closer examination, the hexamers appeared to be true trimers, consisting of three Ty3 capsid dimers.

The examination of fivefold and threefold positions in the particles allowed us to calculate the T-number for each Ty3 particle in the dataset (43). The T-number describes the relative positions of pentamers and hexamers. If pentamers and hexamers are imagined as stepping-stones on the surface of the capsid, then the shortest route between two pentamers consists of h steps in one direction, a 60° turn, and k steps in the new direction: T = h^2^ + hk + k^2^ (*SI Appendix*, Fig. S3). An icosahedral capsid has a single T-number, but some Ty3 particles have mixed T-numbers.

### Two-Dimensional EM of Purified Ty3 Particles.

Cryogrids containing purified PR- Ty3 particles were imaged in a Titan Krios electron microscope equipped with a Falcon II direct electron detector, operated at 300 kV. Images were collected with a nominal magnification of 75,000×, giving a pixel size of 1.08 Å. Images were collected in integrating mode with a total electron dose of 20 electrons/Å^2^. The range of applied defocus values was between −1.0 μm and −3.5 μm.

### Single-Particle Data Analysis.

Ty3 particles were picked manually with eman2 e2boxer (70), resulting in 1,727 individual particles. All further processing steps were performed using RELION software (44). Initially, bin2 data (2.16-Å pixel size) were used for processing to boost computation speed. The particles were extracted and subjected to 2D classification with 12 classes. Noisy and distorted 2D class averages were excluded, and the total number of particles in the dataset was reduced to 1,236. Next, we performed 3D classification into three classes, using a solid sphere as a starting reference. The reconstruction from the most abundant class was used as a reference in subsequent 3D refinement steps. After 28 iterations, the refinement converged, and the measured resolution was 9.2 Å at Fourier shell correlation (FSC) 0.5 and 7.5 Å at FSC 0.143. A Gaussian-smoothened shell mask was used for resolution measurements. The final structure of the Ty3 capsid was B-factor–sharpened (B factor = −400).

### Averaging of Non–Symmetry-Related Subregions.

The Ty3 particles have T = 9, and each asymmetrical unit of the lattice contains nine non–symmetry-related Ty3 capsid protein monomers. We extracted, aligned, and averaged all non–symmetry-related units from the final reconstructions from the two half-datasets (*SI Appendix*, Fig. S6). First, the coordinates of non–symmetry-related units were manually defined using Amira software, and small subboxes were extracted from those positions. Next, several rounds of translational and rotational alignment were performed until convergence. The CA-CTD and the CA-NTD were aligned and averaged separately. The final structure of the CA-CTD comes from nine averaged copies of the domain (Movie S4). Due to differences in the two CA-NTD conformations, only four copies of CA-NTD dimers were averaged together (excluding the CA-NTD from the fivefold position) (Movie S3). The final structure of the CA-CTD was resolved at 4.9-Å resolution at 0.143 FSC, and that of the CA-NTD was resolved at 5.5-Å resolution (*SI Appendix*, Fig. S4). Gaussian-smoothened, ellipsoid-shaped masks were used for resolution measurements.

### Homology Modeling and Fitting.

Homology models of Ty3 CA-CTD and CA-NTD were generated with the use of the HHpred server and Modeler (45, 47). The templates used for homology modeling were the N- and C-terminal lobes of the Arc protein (PDB ID codes 4X3I and 4X3X) (11). The sequence identity between Ty3 and Arc was 10% for CA-NTD and 14% for CA-CTD. The resulting homology models were fitted into the higher resolution EM maps of the CA-NTD and CA-CTD. To improve the quality of the fit, before flexible fitting, the two N-terminal helices of the CA-CTD were repositioned in the density, and the inner loops within the CA-CTD were remodeled separately in Modeler. Similarly the N-terminal helix of the CA-NTD and the inner loops were remodeled. Additionally the very C-terminal tail of the CA-CTD was modeled based on the EM density. They were relaxed into the EM densities with the use of the MDFF package (71). These models were then placed back into the complete EM map of the Ty3 particle. The linker between the CA-NTD and CA-CTD was modeled in Modeler for each of the nine non–symmetry-related positions. The CA-NTD and CA-CTD models were additionally refined in real space in Phenix software (72) before deposition in the PDB.

### Data Availability Statement.

EM maps are deposited in the Electron Microscopy Data Bank, www.ebi.ac.uk/pdbe/emdb (accession codes EMD-4707–EMD-4709) and the PDB, www.ebi.ac.uk/pdbe (ID codes 6R22–6R24).

## Supplementary Material

Supplementary File

Supplementary File

Supplementary File

Supplementary File

Supplementary File

Supplementary File
