# Supplementary material for: Structure of the Ty3/Gypsy retrotransposon capsid and the evolution of retroviruses
Source: Proc Natl Acad Sci U S A. 2019 Apr 29;116(20):10048–57. doi: 10.1073/pnas.1900931116 (PMC6525542; doi:10.1073/pnas.1900931116)
Supplement: Supplementary File [file pnas.1900931116.sapp.pdf]

PNAS
www.pnas.org

Supplementary Information for

The structure of the Ty3/Gypsy retrotransposon capsid and the evolution of retroviruses

Authors: Dodonova S.O.[1,2], Prinz S.[1,3], Bilanchone V.[4], Sandmeyer S.[4], Briggs J.A.G.[1,5,*]

Briggs J. A. G.
Email: john.briggs@mrc-lmb.cam.ac.uk

This PDF file includes:

Supplementary text
Figs. S1 to S9
Captions for movies S1 to S5

Other supplementary materials for this manuscript include the following:

Movies S1 to S5

www.pnas.org/cgi/doi/10.1073/pnas.1900931116

Supplementary Figures

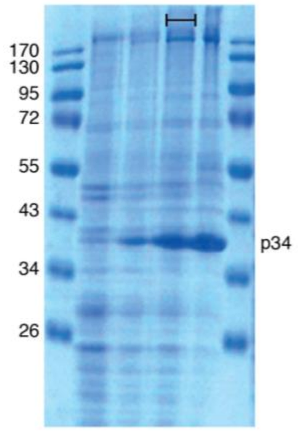

Fig. S1. Ty3 particle purification

Polyacrylamide gel electrophoresis (SDS-PAGE) analysis of sucrose gradient fractions from Ty3 particle purification. The fraction used for subsequent cryo-grid preparation is marked with a black line. A band corresponding to a full-length Gag is clearly visible (p34). No product for a CA (p24) indicates a loss of PR activity.

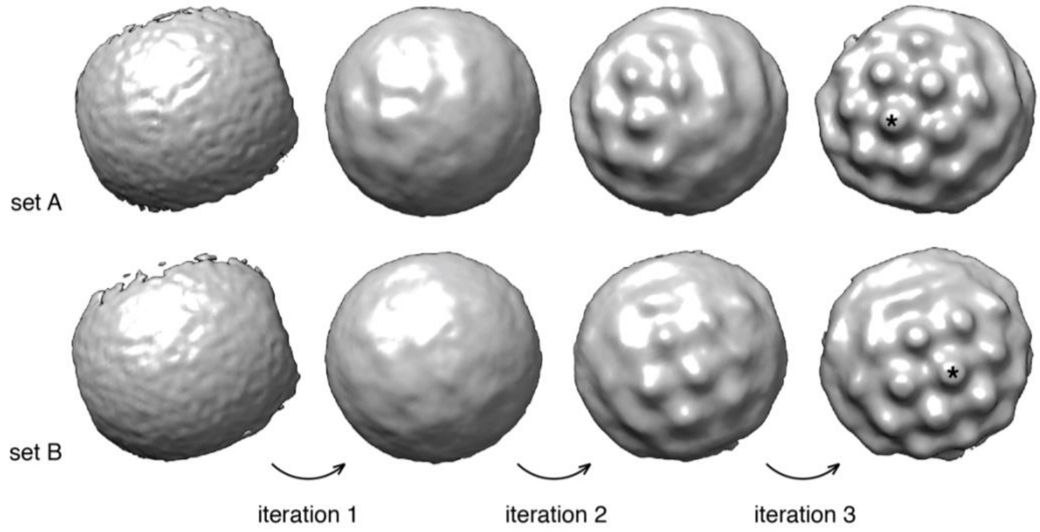

Fig. S2. Initial subtomogram averaging iterations

Evolution of Ty3 capsomer density during subtomogram averaging before any symmetry application. After several iterations the structure shows 6-fold symmetry features.

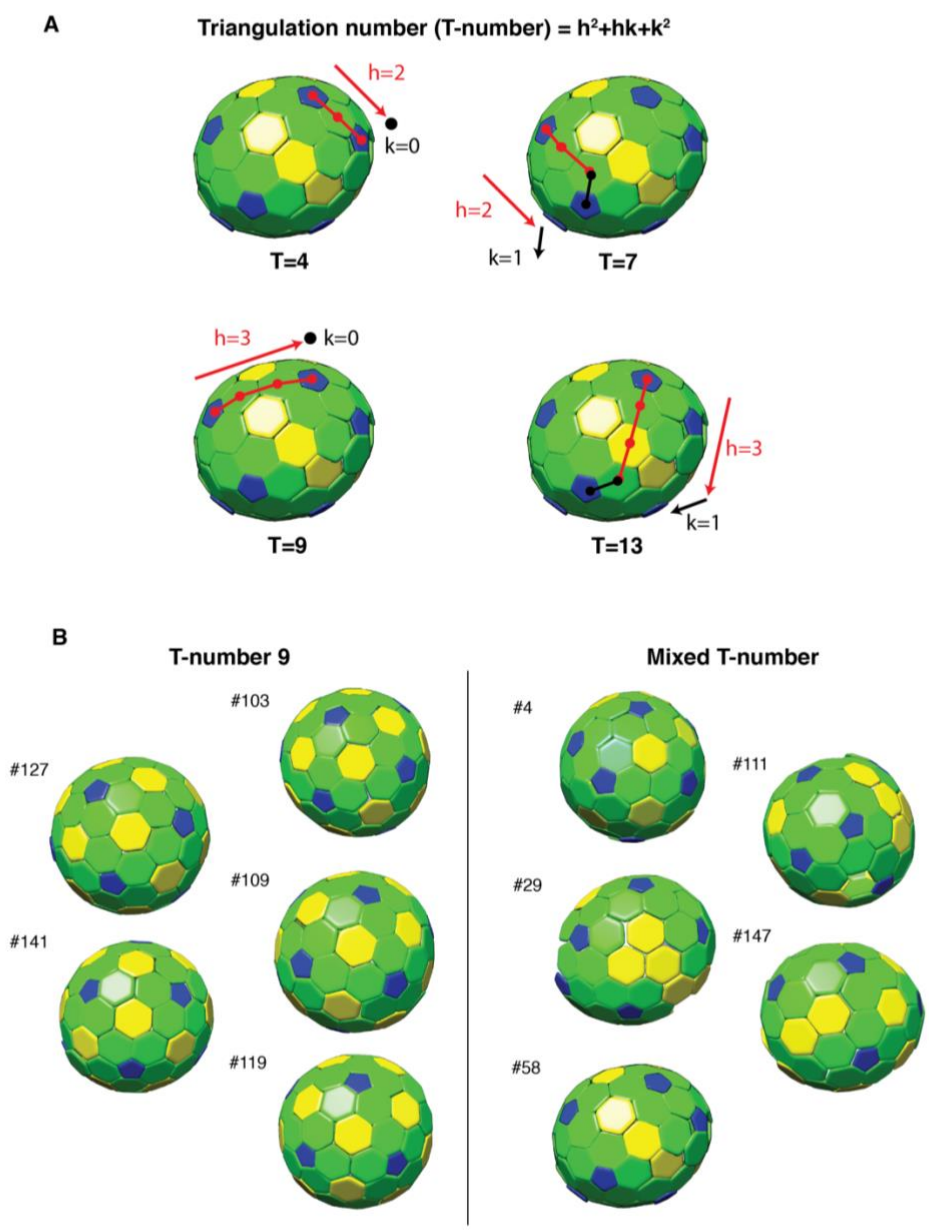

Fig. S3. Ty3 lattice maps

(A) Triangulation (T-numbers) define relative positions of pentamers and hexamers in a capsid lattice. T-number is defined by a formula $T = h^2+hk+k^2$, where the shortest way between two pentamers consists of h steps in one direction, a 60 degree turn, and k steps in the new direction. A regular icosahedral capsid has a single T-number, but irregular capsids can have mixed T-numbers.

(B) Ty3 particle lattice maps: several complete T=9 particles are shown on the left, Ty3 particles with variable T-numbers are shown on the right. Note the irregularity of pentamer positions and the non-spherical shape of particles with variable T-numbers. 5-fold positions are colored in blue, 3-fold in yellow, pseudo-3-fold in green. Numbers indicate sequential numbers of particles in the dataset.

A

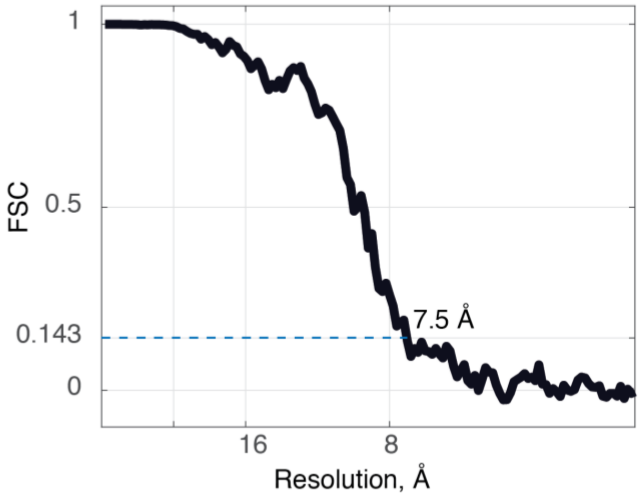

B

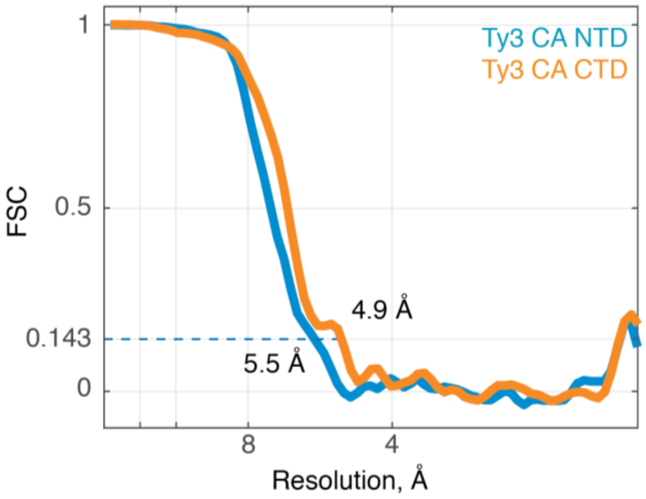

Fig. S4. Assessment of resolution

(A) FSC between two half maps of the complete Ty3 particle. Resolution at the 0.143 cut-off is 7.5 Å. A soft shell-shaped mask was applied to both half maps.

(B) FSC for the high-resolution CA-NTD and CA-CTD. Resolution at the 0.143 cut-off is 4.9 Å for CA-CTD and 5.5 Å for CA-NTD. A soft ellipsoid-shaped mask was applied to both half maps.

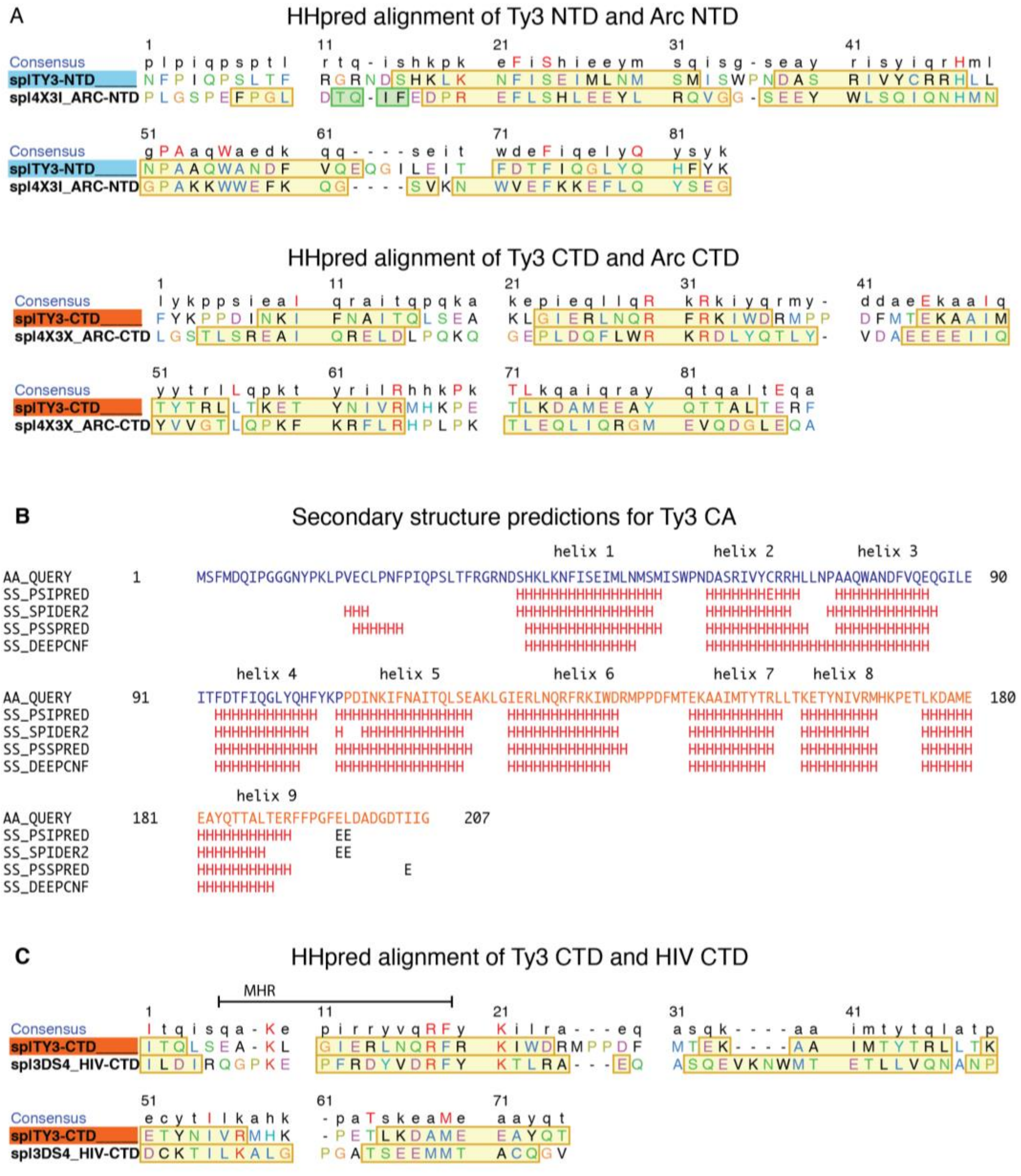

Fig. S5. Secondary structure prediction and sequence alignments

(A) Top: sequence alignment of Ty3 CA-NTD and Arc N-terminal lobe (HHpred). Helices present in the structural models are highlighted by yellow rectangles. Bottom: sequence alignment of Ty3 CA-CTD and Arc C-terminal lobe (HHpred).

(B) Secondary structure prediction for the Ty3 CA (Quick 2D, HHpred server). CA-NTD sequence is colored in dark blue, CA-CTD in orange. “H” shows predicted helical regions.

(C) Sequence alignment of Ty3 CA-CTD and HIV-1 CA-CTD (HHpred). The MHR region is marked with a line.

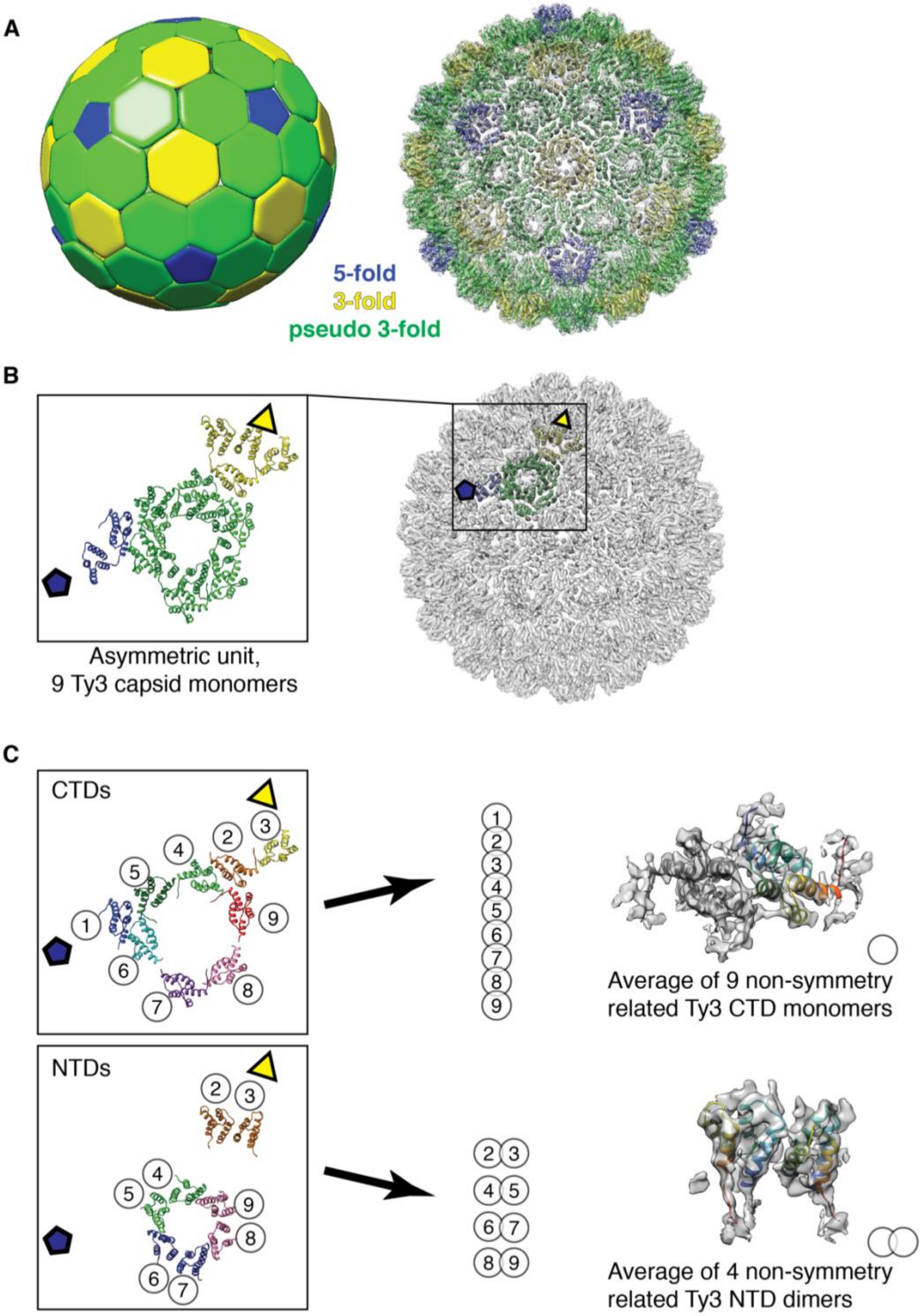

Fig. S6. Averaging of non-symmetry-related subregions

(A) Left: Ty3 T=9 particle lattice map visualized by placing hexagons and pentagons at the positions of capsomers. The 5-fold position is colored in blue, 3-fold in yellow and pseudo-3-fold

in green. Right: a 3D reconstruction of a complete Ty3 particle (EM density in transparent grey) together with the fitted model, with CA molecules colored according to their position in the lattice as in the left panel.

(B) One asymmetric unit of the Ty3 model is shown extracted from the complete model. 5-fold and 3-fold symmetry axes next to the asymmetric unit are marked by a blue pentagon and a yellow triangle respectively. The asymmetric unit contains 9 copies of Ty3-CA.

(C) Schematic representation of the procedure for averaging of non-symmetry related subregions. Top: all 9 non-symmetry related copies of the Ty3 CA-CTD within the asymmetric unit were extracted from the complete EM reconstruction, aligned and averaged together in order to obtain a higher resolution structure. The second copy of the CA-CTD (grey) in the sub-average is included to visualize the CA CTD-CTD interface. See also Movie S4. Bottom: 4 non-symmetry related copies of the CA-NTD dimers were extracted, aligned and averaged together. See also Movie S3.

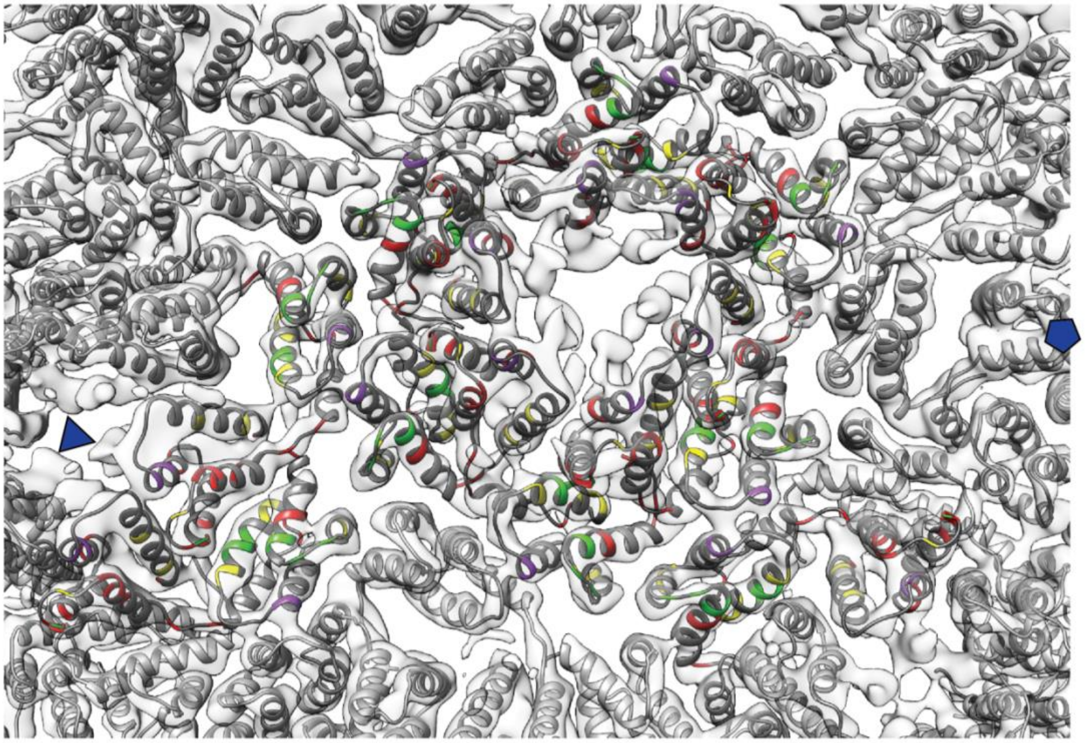

Fig. S7. Ty3 mutation map

Mutations described in (1) are mapped onto the molecular model of the Ty3 capsid. Mutated residues are colored according to the severity of the respective phenotype described in (1): purple – group V (most severe, produced small amounts of Gag3 and were defective by most criteria), red – group IV (no transposition, severe reductions in Gag3 processing, no cDNA), yellow – group III (intermediate transposition phenotype), green – group II (transposition failure, otherwise like WT). Most of the mutated amino acids are located inside of the CA-NTD and CA-CTD and thus would most likely influence domain folding and protein stability and processing. Very few mutated residues are located at the inter-domain interfaces, where they might directly disrupt capsid assembly.

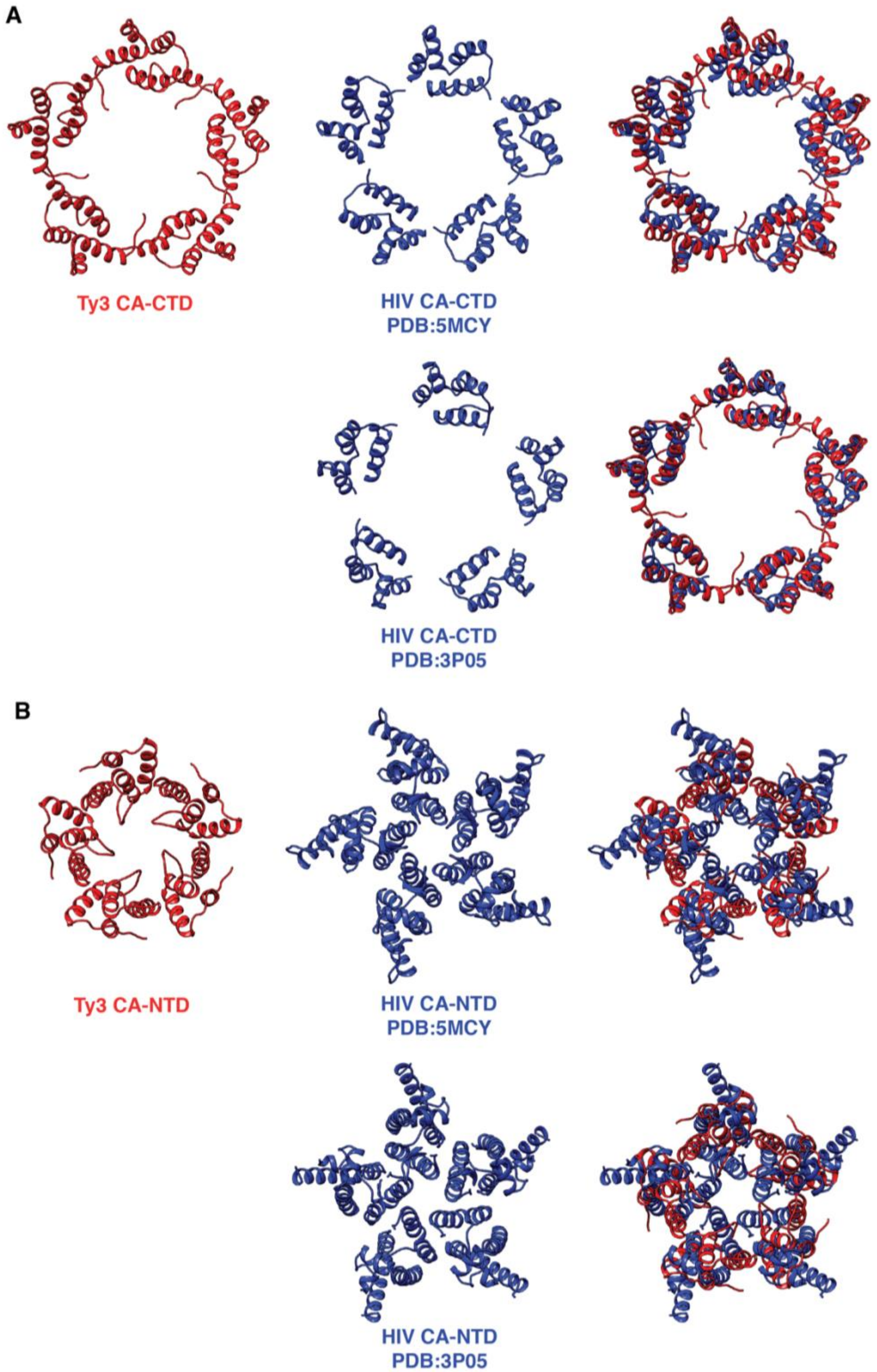

Fig. S8. Structural comparison of the Ty3 and HIV capsid pentamer arrangements

(A) Comparison of the 5-fold CA-CTD quaternary structure of PR- Ty3 with the mature HIV-1 5-fold structure determined by cryo-electron tomography (PDB:5MCY) and the HIV-1 5-fold

structure determined by crystallography (PDB:3P05). The 5-fold CA-CTD structure determined by cryo-electron tomography is slightly expanded relative to that determined by crystallography. The right hand panels show superpositions.

(B) Comparison of the 5-fold CA-NTD quaternary structure of PR- Ty3 with the mature HIV-1 5-fold structures (PDB:5MCY and PDB:3P05). The right hand panels show superpositions. Note the lack of conservation of the CA-NTD quaternary arrangements between HIV-1 and Ty3.

CTD1-NTD2 interface in HIV and TY3

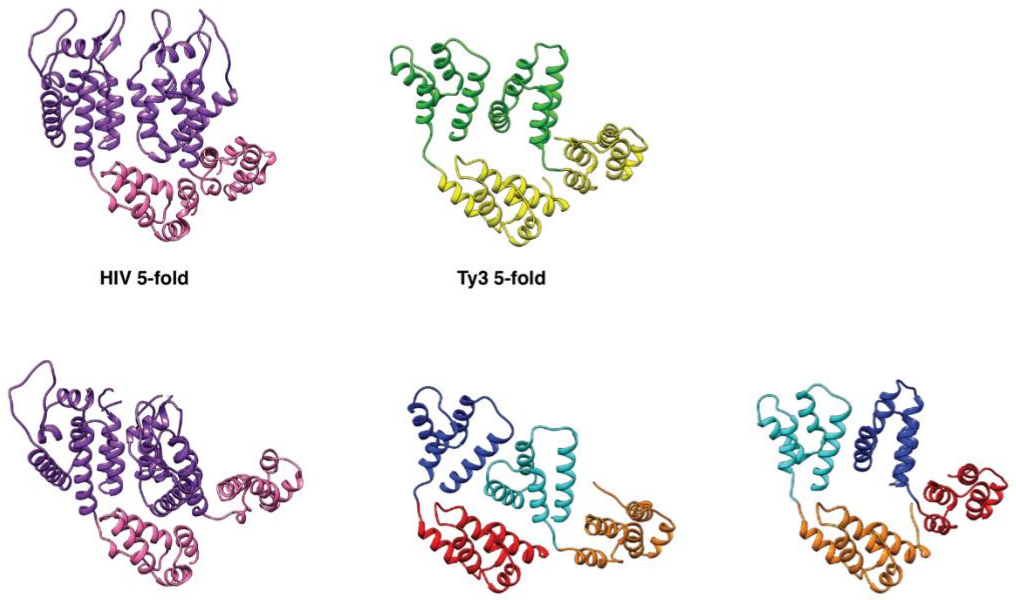

Fig. S9. CA NTD-CTD interface

The interface between the CA-NTD and the CA-CTD in the neighboring CA molecule is shown for HIV-1 5-fold and 6-fold positions, and TY3 5-fold and 3-fold positions. HIV-1 CA-NTD – purple, CA-NTD – pink; Ty3 5-fold CA-NTD – green, CA-CTD – yellow; Ty3 3-fold CA-NTD conformation A/B – cyan/blue, CA-CTD conformation A/B – orange/red. HIV-1 models correspond to PDB IDs: 5MCY (5-fold), 5MCX (6-fold). The interface in HIV-1 6-fold positions is formed by helix 4 and the base of helix 7 in the CA-NTD of one molecule interacting with helix 8 and the top of helix 11 in the CA-CTD of a neighboring molecule. Only the B-A conformation at the 3-fold of Ty3 shows a CA NTD-CTD interface. This involves helices 3 and 4 of the CA-NTD, and helices 6 and 9 in the CA-CTD. These are the equivalent helices to those forming the interface in HIV-1, but the relative orientations and positions of the interacting helices differ.

Movie S1. WT Ty3 tomogram

Tomographic reconstruction of plastic-embedded yeast cells containing WT Ty3 particles. WT type 1 particles have thick-ring morphology, WT type 2 particles have thin ring morphology. Scale bar 100nm.

Movie S2. PR- Ty3 tomogram

Tomographic reconstruction of plastic-embedded yeast cells containing PR- Ty3 particles. Particles are homogeneous, and all have a thick-ring morphology. Scale bar 100nm.

Movie S3. The structure of the CA-NTD determined by averaging of non-symmetry-related copies.

The movie illustrates the averaging of non-symmetry-related CA-NTDs, and the resulting 5.5 Å resolution structure. The structure is also shown at a lower isosurface threshold to reveal density corresponding to the N-terminal part of the protein which is shown as a string of beads. See also Figure 4B and C.

Movie S4. The structure of the CA-CTD determined by averaging of non-symmetry-related copies

The movie illustrates the averaging of non-symmetry-related CA-CTDs, and the resulting 4.9 Å resolution structure. See also Figure 4A.

Movie S5. Ty3 EM map and model

A tour of the Ty3 structure, showing electron density and fitted molecular model.

References

1. Larsen LS, *et al.* (2007) Ty3 capsid mutations reveal early and late functions of the amino-terminal domain. *J Virol* 81(13):6957-6972.
